# Grit as Predictor of Entrepreneurship and Self-Employment in Spain

**DOI:** 10.3389/fpsyg.2019.00389

**Published:** 2019-02-27

**Authors:** Jose L. Arco-Tirado, Ana Bojica, Francisco Fernández-Martín, Rick H. Hoyle

**Affiliations:** ^1^Department of Developmental and Educational Psychology, University of Granada, Granada, Spain; ^2^Department of Business Administration, University of Granada, Granada, Spain; ^3^Department of Psychology and Neuroscience, Duke University, Durham, NC, United States

**Keywords:** big five, specific traits, grit, entrepreneurship, financial resources, self-employment

## Abstract

Extending the growing literature on the role of grit in different life domains, this research explores the relationship between grit and involvement in entrepreneurship. The research highlights the role of personal income and satisfaction with one’s current financial situation as moderators of the relationship between grit and entrepreneurial behavior. Using a large representative sample of Spanish young adults and controlling for a number of potential confounding variables, we find that grit is modestly negatively related to the probability of involvement in entrepreneurship. As predicted, however, this relationship is qualified by both income and satisfaction with current financial situation, though in opposite directions and more weakly for satisfaction with financial status. Gritty individuals with higher levels of income are more prone to become entrepreneurs than gritty individuals with lower levels of income. Gritty individuals with lower levels of satisfaction with their financial situation are more likely to set up a business or become self-employed.

## Introduction

The decision to engage in self-employment/entrepreneurship is influenced by a number of individual characteristics, with a great deal of research focusing on how personality traits can affect the likelihood that people will choose to become self-employed (Chlosta et al., 2012). Increasing attention has been given to the potential influence of grit on individual efforts during episodes of self-employment (Hmieleski and Carr, 2007), owing to the demonstrated prospective association of grit with consequential outcomes that involve persistence in the pursuit of important goals in the educational, personal, and professional domains (Duckworth et al., 2007; Duckworth and Quinn, 2009). Indeed, grit has been shown to predict higher retention among college students (Duckworth et al., 2007); higher educational attainment among adults (Duckworth and Quinn, 2009); greater engagement in the workplace (Eskreis-Winkler et al., 2014); higher labor stability and higher professional efficacy (Duckworth et al., 2007, 2009; Eskreis-Winkler et al., 2014); teacher effectiveness (Robertson-Kraft and Duckworth, 2014); the ability to accomplish strenuous military tasks (Eskreis-Winkler et al., 2014); and individuals’ success operating in contexts characterized by high levels of adversity and setbacks from unexpected events (Westphal et al., 2008). Self-employment brings many of the challenges evident in these endeavors.

The small number of studies about the role of grit in entrepreneurship, though informative, have taken a strictly psychological approach to modeling entrepreneurship, controlling mainly for cognitive factors, traits, and socio-demographic characteristics but ignoring how other structural factors such as socioeconomic background and status might moderate the grit-entrepreneurship relationship. A consideration of such factors is particularly important in the context of entrepreneurship, as the resources entrepreneurs count on to launch a new business, including public policies and programs (Tosun et al., 2016), play a crucial role in the success of the entrepreneurial endeavor and further business development (Baker and Nelson, 2005; Rawhouser et al., 2017). Moreover, some preliminary evidence indicates that structural factors such as socioeconomic status might play an indirect role in the development of grit. For example, lower socioeconomic status children spend less time in extracurricular activities that are related to the development of grit and academic achievement (Park, 2010). In this sense, recent studies have emphasized the need for research on grit to account for the role that structural factors such as socioeconomic origins and status (i.e., the material, social, and cultural resources available to the individual) play in creating the context for grit to support behaviors that help with achievement of challenging long-term goals (Kundu, 2017; Kwon, 2017).

In this research context, our study revolves around the following three key questions: What is the role of grit in predicting attempted entrepreneurship/self-employment? How do available financial resources affect the probability of a gritty individual starting a business? And finally, how does satisfaction with one’s financial situation affects the relationship between grit and the probability of creating a business/becoming self-employed? Below, we develop the arguments supporting our research questions that lead us to our hypotheses.

### Individual Differences and Entrepreneurship

The individual difference perspective involves different areas of research such as career perspective, personality differences, health and well-being, cognition and behavior, and entrepreneurial leadership (Gorgievski and Stephan, 2016; Mannino and Faraci, 2017). It encompasses two dominant research approaches to the study of entrepreneurship: the competency approach and the personality approach.

The competency approach focuses on an integrated combination of knowledge, skills, and attitudes that can be changed, learned, and attained through experience, training, or coaching (e.g., ability to plan ahead, orientation toward learning, ability to identify and seize opportunities for success, taking risk, perseverance, decisiveness, independence, ability to persuade, self-knowledge, self-confidence (Kyndt and Baert, 2015). Drawing on work from this perspective to understand entrepreneurs’ activities and actions at an intra-individual level, i.e., focusing on the so-called micro-foundations of entrepreneurship, psychological scientists have an opportunity to make significant contributions to our understanding of entrepreneurial behavior (Frese, 2009).

The personality approach to the study of entrepreneurship may take two primary forms (Muñiz et al., 2014). A significant number of studies have focused on identifying those general personality factors such as the Big Five that have a clear relationship with entrepreneurship. Early reviews of relevant work raise question as to the relationship between Big Five traits (e.g., conscientiousness, openness) and entrepreneurial success (e.g., Gartner, 1985). It appears that the personality approach in this form conceptualizes traits too broadly for them to predict the specific behaviors characteristic of entrepreneurial activity (Suárez-Álvarez et al., 2014). Furthermore, work based on this form of the personality approach has produced contradictory results (Sánchez et al., 2011).

More recent studies (e.g., Zhao and Seibert, 2006; Rauch and Frese, 2007; Zhao et al., 2010) have found that an alternative form of the personality approach focused on narrow personality traits (e.g., facets) offers better prediction of entrepreneurial behavior than broad traits (Mooradian et al., 2016). These studies highlight the value of focusing more on specific personality characteristics such as innovativeness, proactive personality, and self-efficacy, which have a significant and positive relationship with entrepreneurial success, or autonomy and internal locus of control, which predict both firm creation and entrepreneurial success (e.g., Rauch and Frese, 2007; Suárez-Álvarez et al., 2014). Indeed, research syntheses indicate that allowing for matching of relevant traits to the task characteristics of entrepreneurs yields stronger results. For example, self-efficacy, proactive personality, and achievement motivation correlate more highly with business creation and success than do all other factors, including the Big Five traits and human and social capital (Frese and Gielnik, 2014).

Other studies of enterprising individuals from the individual differences perspective have taken a cognitive approach (e.g., Sánchez et al., 2011). Based on general mental ability and creativity research, these studies have sought to explain how people process information, that is, how people comprehend and make associations between information in the context of entrepreneurship. More recently, this corpus of research has put specific emphasis on cognitive capacities such as entrepreneurial alertness and abilities to detect opportunities or make quick decisions under conditions of uncertainty and limited time (Shane, 2004; Frese and Gielnik, 2014; Mooradian et al., 2016; Wolfe and Patel, 2016).

### Grit as a Narrow Trait

In addition to the nine narrow traits identified in the entrepreneurship literature (i.e., achievement motivation, risk taking, innovativeness, autonomy, self-efficacy, stress tolerance, internal locus of control, external locus of control, and optimism) (Suárez-Álvarez et al., 2014), recent studies have considered the potential of grit to strengthen the capacity of current models to predict entrepreneurial behavior. Grit, defined as “perseverance and passion for long-term goals,” is associated with an individual’s ability to put forth sustained effort to achieve challenging goals, particularly in the face of trials and adversity (Duckworth et al., 2007; Borghans et al., 2008; Duckworth and Yeager, 2015). In the context of broad models of personality, grit aligns most closely with the conscientiousness domain (Roberts et al., 2014; Rimfeld et al., 2016; Arco-Tirado et al., 2018) as reflected in its conceptual relatedness to conscientiousness facets such as orderliness, dependability, self-control, and industriousness (Duckworth and Eskreis-Winkler, 2013). Yet, despite its conceptual similarity to facets of conscientiousness, none of the conscientiousness facets fully captures the combination of passion and perseverance that characterizes grit. In particular, the sustained interest in important long-term goals, a core feature of grit, is not evident in conscientiousness or its facets. Thus, although the conceptual overlap between the broad conscientiousness domain and the relatively narrow grit trait is substantial, conceptually speaking, grit includes a specific and unique focus on the pursuit of long-term, higher-order goals (Duckworth and Quinn, 2009). Whether or not grit overlaps with or is subordinate to conscientiousness, it has been demonstrated to offer important explanatory power in variables related to long term tenacity and passion for goals across time—a distinctive capacity that is likely to explain success in the entrepreneurial context (Mooradian et al., 2016).

## Hypotheses

### Grit as Fuel for Entrepreneurship/Self-Employment

Recently, the influence of grit has also been explored with respect to entrepreneurship, understood as self-employment (Wolfe and Patel, 2016) or the creation of a new business (Mooradian et al., 2016; Mueller et al., 2017). In a sample of the general population from developing countries, Wolfe and Patel (2016) found that grit is related to self-employment, but also that grit is the most strongly related to self-employment for risk-takers, women, and younger adults. The findings are robust, holding after controlling for several individual characteristics and alternative explanations. Specifically, these authors suggest that grit is a more important factor in determining self-employment for individuals with higher risk-taking propensities than it is for individuals with lower risk-taking propensities because of the higher levels of failure and therefore frustration they are likely to face; for females more than males due to the substantially more hindrances that women have to face in the path to becoming self-employed; and, finally, for younger more than older individuals because of their difficulties accessing the necessary resources in the entrepreneurial process.

The hypothesized underlying mechanism for the relationship between grit and the decision to engage in entrepreneurship is, according to Wolfe and Patel (2016), the increased self-confidence gritty individuals develop. These authors base this argument on previous results of Kolvereid and Isaksen (2006) and Verheul et al. (2012), who found that the self-perceived ability to succeed depends on people’s confidence in their ability to overcome adversities. This confidence could result from the role of grit as fuel for people to persevere in their efforts toward developing the skills, knowledge, and competencies necessary to judge whether they have the ability to succeed if they engage in self-employment (Wolfe and Patel, 2016). So, these arguments lead us to formulate hypothesis 1: Grit is associated with a higher probability of creating a business/becoming self-employed during young adulthood.

### Personal Financial Resources

Because not all individuals with such personality traits and characteristics will start a new business, it is useful to ask what makes some people, but not others, with such specific personality traits go on to engage in entrepreneurship (Thompson, 2009). Thus, although there has been substantial research into the influence of personal characteristics (i.e., perceived ability) on engagement in self-employment (Kolvereid and Isaksen, 2006), no previous studies have focused on the role of other personal factors (e.g., personal monthly net income level, satisfaction with personal financial situation) as moderators of the association between grit and entrepreneurship/self-employment. Previous research indicates that grit contributes to the motivation to engage in entrepreneurial behavior, and that this relationship is moderated by different socio-demographic variables such as gender and age (Wolfe and Patel, 2016). These findings suggest that those who face more barriers in the entrepreneurial process and have more resource restrictions (i.e., women and young individuals) are the ones who benefit most from grit (Meriac et al., 2015). However, prior research in the entrepreneurship field on resilient behaviors such as bricolage (i.e., making-do with resources at hand; Baker and Nelson, 2005) has found that even though these resilient behaviors can help people face adversity, they can be displayed—and in fact are displayed most strongly—in less adverse conditions (Desa and Basu, 2013; Bojica et al., 2018). For example, Baker and Nelson (2005) emphasized that, whereas making do with resources at hand helps entrepreneurs acting in impoverished circumstances to create the resources they need and respond to different challenges in the entrepreneurial process, this resilient behavior is likely to inhibit the growth of their organization by dispersing the effort to multiple bricolage subprojects. Conversely, when bricolage is applied in organizations with high levels of resource endowments as a mean to stimulate the creative use of resources, it leads to organizational growth (Bojica et al., 2018). These findings suggest that traits like grit, which help individuals make progress in spite of adversity, are likely be even more beneficial when displayed in favorable conditions.

In this vein, the resources entrepreneurs count on to launch a new business, including public policies and programs (Tosun et al., 2016), play a crucial role in the success of the entrepreneurial endeavor and further business development (Baker and Nelson, 2005; Rawhouser et al., 2017). In a sample of nascent American entrepreneurs, Gartner et al. (2012) found that entrepreneurs’ personal contributions represent 57.34% of all financing used. Therefore, an entrepreneur’s current financial resources are likely to be an important factor that motivates or enables entrepreneurial behavior. Moreover, some preliminary evidence indicates that structural factors such as socioeconomic status might play an indirect role in the development of grit. For example, lower socioeconomic status children spend less time in extracurricular activities that are related to the development of grit and academic achievement (Park, 2010). In this sense, recent studies have emphasized the need for research on grit to account for the role that structural factors such as socioeconomic origins, status (i.e., the material, social, and cultural resources available to the individual) play in creating the context for grit to support behaviors that help with achievement of challenging long-term goals (Kundu, 2017; Kwon, 2017). Specifically, we propose that people who can count on higher levels of personal income will have more resources for supporting themselves in the entrepreneurial process, and this will allow them to concentrate their effort on the challenges intrinsically associated with the entrepreneurial process, thereby enhancing the relationship between grit and the probability of creating a business/becoming self-employed. Consistent with this proposal, prior research on self-employment and income distribution shows that the population of self-employed individuals concentrates in the lower and upper tails of the income distribution (Hamilton, 2000). So, these arguments lead us to formulate hypothesis 2: The relationship between grit and the probability of creating a business/becoming self-employed will be more strongly positive for young adults the greater their personal financial resources.

### Satisfaction With Own Financial Situation

Grit is a predictor of life satisfaction (Singh and Jha, 2008). In the present research we explore the role of satisfaction, and particularly satisfaction with one’s financial situation, as a factor that might stimulate grit in service of entrepreneurial outcomes. We propose that gritty individuals who are less satisfied with their financial situation will be more prone to be involved in entrepreneurship, mainly because of the following reasons.

First, people who are less satisfied with their economic situation may perceive a higher level of adversity and this may make grit even more important for persisting in the entrepreneurial endeavor. In general, previous studies offer significant evidence of the value of grit for overcoming adversity particularly in relation to involving in self-employment (Wolfe and Patel, 2016).

Second, studies from the disadvantage theory of entrepreneurship (Light, 1979) indicate that some people who engage in entrepreneurship are misfits cast-off from the wage work. Specifically, these studies show that workers having a lower wage and, therefore, those who are more likely to be dissatisfied with their financial situation, are also more likely to enter self-employment or be self-employed at a some point in time (Evans and Leighton, 1989; Acs and Audretsch, 1990). In the same vein, other studies find that most of the individuals starting a business are unemployed (e.g., Evans and Leighton, 1989; Carrasco, 1999; Millan-Tapia, 2013). The present study excludes from the analysis those individuals who started a business out of necessity, because the drivers, circumstances, and mechanisms at play in the decision to become self-employed/start a business are likely to be different than in the case of those who are pushed versus pulled into entrepreneurship. We propose that, within the group of individuals that are not excluded from the traditional labor market, those who are not satisfied with their economic situation will be the ones more motivated to explore the path of self-employment/creating a business, as dissatisfaction with one’s economic situation might lead the individual to explore new avenues for generating income.

Moreover, previous research shows that occupational expectations are less consistent in time particularly when people’s socioeconomic background is lower (Rindfuss et al., 1999). Therefore, we would expect people who are less satisfied with the outcomes of their occupational choices to be more prone to change and explore other alternatives. This situation can lead gritty individuals to dedicate more time and effort to explore alternative options to generate income, strengthening the relationship between grit and entrepreneurship.

Taken together, these arguments lead us to formulate the following hypothesis 3: The relationship between grit and the probability of creating a business/becoming self-employed will be more strongly positive for young adulthoods the less satisfied they are with their own financial situation.

## Materials and Methods

The data used in this study were collected as part of a large-scale research project on the cultural pathways to economic self-sufficiency and entrepreneurship and the role of family values in 11 European countries (CUPESSE). Data were collected through a survey administered to young adults in each of the 11 countries. We used the subsample of young adults from Spain; by using data from a single country, we avoided the need to consider cultural differences in the initial tests of our hypotheses.

### Participants

The sample included 1004 participants between 18 and 35 years old, with an average age of 27.44 years old (*SD* = 4.91), and equivalent numbers of women (*n* = 510, 50.8%) and men (*n* = 494, 49.2%). A proportional stratified random sampling technique was used with regions (i.e., Nomenclature of Units for Territorial Statistics-2), employment status, age and sex working as strata. Nearly three-quarters of the sample (71.5%) had relatively high levels of education (i.e., upper tier upper secondary education or higher) and a bit more than a half (i.e., 55.08%) was employed or self-employed.

### Materials

The CUPESSE survey focuses on economic self-sufficiency, employability, entrepreneurship, and the family transmission of traits and attitudes that affect such outcomes. The survey was fielded in Spain in 2016 after translation into Spanish by the University of Granada CUPESSE partner following recommendations in the Cross-Cultural Survey Guidelines, adhering closely to the TRAPD (Translation, Review, Adjudication, Pretesting, and Documentation) team translation model (Survey Research Center, 2016; see Arco-Tirado et al., 2018 for more information).

### Measures

#### Dependent Variable

Entrepreneurship was measured by a single item asking respondents, “Have you ever started your own business/become self-employed?” It was coded as a dichotomous variable with the values 1 = *Yes* and 0 = *No*.

#### Control Variables

A number of variables were used as regression control variables, as informed by previous empirical work (Brandstäter, 2011; Frese and Gielnik, 2014; Gorgievski and Stephan, 2016; Wolfe and Patel, 2016). *Sex* was coded dichotomously, with 1 indicating *male* and 2 indicating *female*. *Age* (was measured in years. Respondents indicated their *Level of Education* (“What is the highest level of education you have successfully completed?”) by choosing one of seven response options: 1 = *ES-ISCED I, less than lower secondary*, 2 = *ES-ISCED II, lower secondary*, 3 = *ES-ISCED IIIb, lower tier upper secondary*, 4 = *ES-ISCED IIIa, upper tier upper secondary*, 5 = *ES-ISCED IV, advanced vocational, sub-degree*, 6 = *ES-ISCED V1, lower tertiary education*, and 7 = *ES-ISCED V2, higher tertiary education < = MA level*. Values were re-coded as *basic education, secondary education*, or *superior education*; basic education was used as the reference group in regression models. *Previous work experience* (“Have you ever had a paid job for 1 year or more?”) was coded dichotomously, with 1 indicating *Yes* and 2 indicating *No*. Respondents rated their *risk-taking* (“On a scale from 0 to 10 would you say that in general you are a person who tends to avoid taking risks or are you fully prepared to take risks?”) on a Likert-type scale with values ranging from 0 = *tend to avoid risks* to 10 = *I am fully prepared to take risks*. *Self-efficacy* (“I am confident that I can deal efficiently with unexpected events”), *personal initiative* (“Usually I do more than I am asked to do”), and *locus of control* (“My life is determined by my own actions”) were rated on a Likert-type scale with the following values: 1 = *Strongly disagree*, 2 = *Somewhat disagree*, 3 = *Somewhat agree*, and 4 = *Strongly agree*. *Born in the country* (“Were you born in [country]?”) variable was measured based on the ISO 3166-1 numeric classification. *Financial situation when 14* (“Financial situation: My family was able to pay its bills”) was indicated on a scale with from the following response options: 1 = *Never*, 2 = *Sometimes*, 3 = *Most of the time*, and 4 = *Always*.

#### Independent Variables

*Grit* was measured using the Spanish version (Arco-Tirado et al., 2018) of the original short Grit Scale (Grit-S) developed by Duckworth and Quinn (2009) using a Likert-type scale with the following values 1 = *Strongly disagree*, 2 = *Somewhat disagree*, 3 = *Somewhat agree*, and 4 = *Strongly agree*. For analysis purpose item values were reversed for items 1, 2, 3, 5, and 6. Internal consistency estimate was acceptable: α = 0.75 for overall grit. *Personal monthly total net income* (“If you add up the income from all sources, which number describes your personal monthly total net income?”) was measured with a Likert-type scale with values ranging from 1 = *Lowest* to 10 = *Highest*. *Satisfaction with financial situation* (“Thinking about your own financial situation, how satisfied are you right now?”) was assessed using a Likert-type scale with the following values 1 = *Very dissatisfied*, 2 = *Rather dissatisfied*, 3 = *Rather Satisfied*, and 4 = *Very satisfied*.

### Procedure

Once the translation of the survey was completed, the implementation process comprised two steps. First, the polling firm in collaboration with the research team generated a probability sample of young adults and surveyed them with questions from the “Youth Questionnaire.” Second, all respondents were asked for the contact of one or both parents, and who were surveyed using questions from the “Parental Questionnaire.” The fieldwork was undertaken by a polling company operating in Spain (i.e., NetQuest, MDK). The aim was to reach a net minimum of 1.000 surveys from young adults. Regarding the young adults’ data, the survey company was asked to provide a probability sample of young adults between 18 and 35 years old, representative for employment status (e.g., employed; self-employed; unemployed; in education/training), NUTS-2 region, age categories, education, and migration background/minority group membership in Spain. The survey technique followed was the Computer Assisted Telephone Interviewing (CATI). NetQuest panel is recruited “by invitation only” using hundreds of websites with validated databases. This multisource approach results in a broad range of socio-demographic profiles. Thus, prospective respondents were sent an online invitation and given 14 days to respond. The invitation to panel members provided information on the objectives of the research, the voluntary nature of their participation, and the confidentiality of their responses. Panel members who elected to participate were provided a respondent-specific link to access the Spanish version of the youth questionnaire. The sampling frame used by the polling firm consisted of 113,739 young adults; the response rate was.205%. Questionnaire pre-tests were conducted between December 2015 and February 2016. Insights from pre-tests produced led to minor changes in the questionnaire. Responses for analysis were collected from February to June of 2016.

### Statistical Analyses

We tested our hypotheses using binomial logistic regression analysis, which estimates and tests the probability of an event occurring. We used maximum likelihood estimation, which is a robust method for dealing with data that are not normally distributed (Steenkamp and van Trijp, 1991).

As it can be seen in Table 2, we ran the analyses in three steps. In the first step we introduced as predictors only the control variables. In the second and third steps we added the effect of the independent variables and the interaction effects, respectively. For each variable, Table 2 shows the beta coefficient, its level of significance, and the odds ratios (within brackets). For each model, it also presents the values of the Wald chi-square, Log likelihood, Cox and Snell *R*^2^, Nagelkerke *R*^2^, and the Hosmer–Lemeshow test, which are used to assess model goodness of fit.

## Results

The results of the correlation and descriptive analysis of all the variables included in the study are presented in Table 1. The modest values of the correlation coefficients indicate that multicollinearity should not be a problem in estimating our models (Tabachnick and Fidell, 2001). Because the table includes covariates and moderators, a subset of the correlations are not expected to be significant. Nonetheless, more than half of the coefficients are statistically significant. Their magnitude generally is small, owing largely to the fact that many of the variables are multiply determined and thus only modestly related to any one of the multiple determinants.

**Table 1:** Means, standard deviations, and correlations.

	Mean	*SD*	1	2	3	4	5	6	7	8	9	10	11	12	13	14
(1) Sex	1.51	0.500														
(2) Age	27.44	4.910	0.035													
(3) Secondary education	0.22	0.415	-0.035	-0.317^**^												
(4) High education	0.57	0.494	0.069^*^	0.314^**^	-0.629^**^											
(5) Previous work experience	1.44	0.496	-0.070^*^	-0.579^**^	0.238^**^	-0.188^**^										
(6) Risk-taking	5.54	2.509	0.018	-0.078^*^	0.014	0.034	0.004									
(7) Self-efficacy	3.38	0.630	0.065^*^	0.094^**^	-0.033	0.066^*^	-0.146^**^	0.228^**^								
(8) Personal initiative	3.28	0.679	0.153^**^	0.163^**^	-0.066^*^	0.083^**^	-0.155^**^	0.129^**^	0.367^**^							
(9) Locus of control	3.27	0.702	0.028	-0.034	0.003	0.059	-0.029	0.086^**^	0.187^**^	0.179^**^						
(10) Born in the country	1.06	0.232	0.044	-0.025	0.026	-0.076^*^	0.006	0.070^*^	-0.006	0.002	-0.030					
(11) Financial situation when 14	3.00	0.674	0.051	-0.039	0.029	0.136^**^	0.041	0.019	-0.028	0.020	0.050	-0.162^**^				
(12) Personal monthly total net income	2.82	2.248	-0.019	0.255^**^	-0.097^**^	0.167^**^	-0.323^**^	-0.063	0.079^*^	0.064	-0.004	-0.052	0.084^*^			
(13) Satisfaction with financial situation	2.17	0.805	0.028	-0.017	0.000	0.062	-0.156^**^	-0.005	0.021	-0.011	0.026	-0.054	0.170^**^	0.443^**^		
(14) Grit	2.87	0.485	0.153^**^	0.140^**^	-0.064^*^	0.133^**^	-0.079^*^	0.094^**^	0.293^**^	0.254^**^	0.116^**^	0.047	0.029	0.062	0.044	
(15) Started own business/become self-employed	0.11	0.319	0.005	0.097^**^	-0.062	0.041	-0.133^**^	0.037	0.046	0.042	0.051	0.034	0.009	0.032	-0.071^*^	-0.028


*^∗^*p* < 0.05; ^∗∗^*p* < 0.01; ^∗∗∗^*p* < 0.001.*

Table 2 presents the results of the binomial logistic regression analysis. Model 1 presents the effects of the control variables alone. The number of observations available for analysis is 926 because of missing values for some of the variables used in the logistic regression and because 37 the cases of entrepreneurship/self-employment by necessity (as opposed to choice) were excluded from the analysis. The Wald chi-square is statistically significant and, together with the lack of significance of the Hosmer–Lemeshov test, indicates that overall the model fits the data. Among the control variables, only previous work experience has a significant association with the probability of creating a firm or becoming self-employed. Young people who have previous work experience are more likely to create a business. The odds of creating a business is 0.535 lower for people who had no significant previous work experience (1 year or more as employed). This finding is in line with previous entrepreneurship studies, according to which young people with more work experience are more prepared to create a business (Shane, 2004).

**Table 2:** Results of the binomial logistic regression analysis.

Predictor	Model 1	Model 2	Model 3a	Model 3b
Sex	-0.073 (0.929)	-0.0344 (0.957)	-0.024 (0.976)	-0.036 (0.965)
Age	0.020 (1.020)	-0.014 (0.986)	-0.014 (0.986)	-0.013 (0.988)
Secondary education	-0.365 (0.694)	-0.211 (0.810)	-0.272 (0.762)	-0.207 (0.813)
High education	-0.086 (0.917)	-0.075 (0.928)	-0.076 (0.926)	-0.081 (0.922)
Previous work experience	-0.766^∗∗^ (0.465)	-0.926^∗∗^ (0.396)	-0.933^∗∗^ (0.394)	-0.918^∗∗^ (0.399)
Risk-taking	0.042 (1.042)	0.037 (1.037)	0.039 (1.040)	0.040 (1.041)
Self-efficacy	0.069 (1.071)	0.196 (1.217)	0.191 (1.211)	0.207 (1.230)
Personal initiative	0.027 (1.028)	0.159 (1.172)	0.194 (1.214)	0.154 (1.167)
Locus of control	0.225 (1.253)	0.182 (1.200)	0.167 (1.182)	0.176 (1.192)
Born in the country	0.522 (1.685)	0.659 (1.933)	0.658 (1.931)	0.668 (1.950)
Financial situation when 14	0.090 (1.095)	0.135 (1.145)	0.124 (1.132)	0.123 (1.131)
Personal monthly total net income (PMI)		0.096 (0.101)	-0.530^†^ (0.589)	0.095 (1.100)
Satisfaction with financial situation (SFS)		-0.607^∗∗^ (0.545)	-0.615^∗∗^ (0.541)	-2.063^∗^ (0.127)
Grit		-0.563^∗^ (0.569)	-1.213^∗∗^ (0.297)	-1.583^∗^ (0.205)
Grit ^∗^ PMI			0.216^∗^ (1.241)	
Grit ^∗^ SFS				0.509^†^ (1.663)
Constant	-3.503^∗^ (0.030)	-0.845 (0.430)	0.995 (2.704)	2.011 (7.474)
Wald chi square	396.164^∗∗∗^	321.849^∗∗∗^	321.849^∗∗∗^	321.849^∗∗∗^
Log likelihood	617.884	485.407	481.174	482.275
*R*^2^ Cox and Snell	0.026	0.042	0.048	0.046
*R*^2^ Nagelkerke	0.052	0.085	0.096	0.093
Hosmer–Lemeshow	13.594^†^	7.448 (n.s.)	8.327 (n.s.)	2.763(n.s.)
% Correct predictions	89.0	89.1	89.1	89.1


*Coefficients associated with hypotheses are Grit, Grit^∗^PMI, and Grit^∗^SFS. ^†^*p* < 0.10; ^∗^*p* < 0.05; ^∗∗^*p* < 0.01; ^∗∗∗^*p* < 0.001.*

Model 2 add variables involved in the focal interaction effects. Goodness of fit for the model is comparable to that of Model 1. Both grit and degree of satisfaction with financial situation are associated with starting a business. Contrary to our prediction, grit is significantly and negatively related to becoming an entrepreneur. Satisfaction with financial situation is also negatively related to becoming an entrepreneur. Personal monthly total net income is not associated with the starting a business.

**FIGURE 1:**
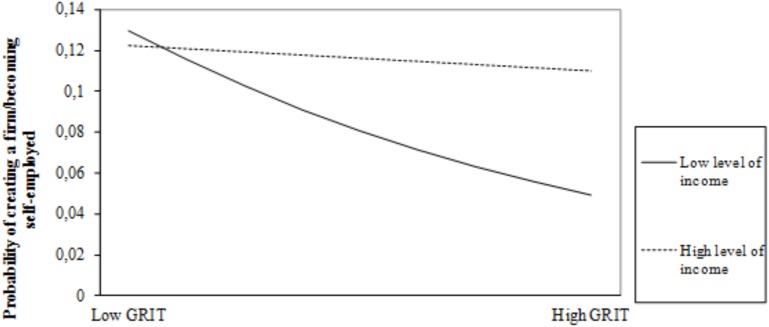
Moderating effect of level of income on the relationship between grit and entrepreneurship/self-employment.

**FIGURE 2:**
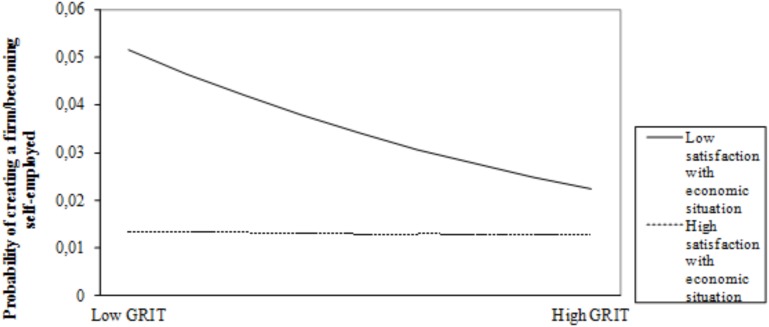
Moderating effect of satisfaction with own economic situation on the relationship between grit and entrepreneurship/self-employment.

Models 3a and 3b include the interaction effects between grit and level of income and grit and satisfaction with own economic situation, respectively. Like in the previous models, the statistics show good fit between model and data and the explanatory power of the models increases, as shown by the pseudo *R*^2^ represented by Cox and Snell *R*^2^ and Nagelkerke *R*^2^, which improve over the corresponding values for Model 2. Whereas the beta coefficient for the interaction term between grit and level of income is positive and significant, the coefficient of the interaction term between grit and satisfaction with own financial situation is negative and marginally significant. As expected, individuals who score higher on grit are more prone to become entrepreneurs when they can count on higher levels of financial resources than when they have lower levels of economic resources (see Figure 1). And, as predicted, a lower degree of satisfaction with own financial situation will fuel the entrepreneurial initiative of gritty individuals (see Figure 2). These results offer support for our second hypothesis and marginal support for our third hypothesis.

## Discussion

This study explored the role of grit as a function of objective and self-perceived financial situation in determining whether young adults start a new business/become self-employed. Although the effect was weak, when controlling for a host of potential confounding variables, we observed a negative simple relation between grit and entrepreneurship, an outcome that is inconsistent with our hypothesis. Additionally, and as expected, we found that this relationship is moderated by objective and subjective features of young adults’ financial situation. Specifically, we found significant support for the hypothesis that young adults with higher levels of income and grit are more prone to take the step into entrepreneurship/self-employment than individuals with lower levels of income and high levels of grit. We also found marginal support for the hypothesis that a low satisfaction with one’s financial situation and high levels of grit make individuals more prone to engage in entrepreneurship than when they are satisfied with their economic situation and high in grit. We suggest several plausible explanations for these findings.

Regarding the relationship between grit and entrepreneurship, the baseline argument of previous research and our hypothesis was that grit leads young adults to display more self-confidence and perseverance (Hmieleski and Carr, 2007; Wolfe and Patel, 2016), which are positively associated with entrepreneurship. Therefore, gritty individuals would be more prone to engage in entrepreneurship. However, these capabilities and behaviors are not exclusive to entrepreneurship and can be displayed in other occupational alternatives.

The nature of our sample–young adults (below 35 years old) excluding those that started a business out of necessity—suggests a relevant explanation of the results obtained. On one hand, individuals’ motivations and aspirations during this life stage might prioritize other occupational choices like employment or continue education. In fact, previous research indicates that grit is associated with the achievement of higher levels of education (Duckworth and Quinn, 2009), engagement with the workplace, and higher labor stability (Duckworth et al., 2007; Eskreis-Winkler et al., 2014). Therefore, gritty individuals might just stay longer in education and search for more stable employment opportunities.

On the other hand, the structure of economic incentives does not appear to make entrepreneurship/self-employment a better option than employment. Previous studies both in the US (e.g., Carrington et al., 1996; Hamilton, 2000) and European countries (Millan-Tapia, 2013) comparing earnings from employment and self-employment indicate that the former provides higher levels of income than the latter. If to the low economic incentives we add the high levels of uncertainty entailed by self-employment, the latter may not be *a priori* a desirable career choice (Moskowitz and Vissing-Jorgensen, 2002), particularly in countries with high levels of uncertainty avoidance such as Spain. In this sense Kolvereid (1996) emphasized that security is the most important reason for individuals’ preferences between organizational employment and self-employment. Our finding with respect to satisfaction with one’s economic situation seems to support the idea that entrepreneurship is taken as a career choice when the level of income obtained from other career alternatives is not considered satisfactory.

Even though young adults might not be aware of the probability of earning less by being self-employed, or they might be overly optimistic regarding the potential revenue of their business idea, they have less human, financial, and social capital than older adults. Lack of adequate resources constitutes an important obstacle to the establishment of a new business and persistence in the entrepreneurial endeavor (e.g., Bates, 1995; Gimeno et al., 1997; Davidsson and Honig, 2003). Available data indicate that indeed the entrepreneurship rate among young people is lower than among older adults (Red GEM España, 2017). From this perspective, even if young gritty individuals would see entrepreneurship as an attractive career opportunity, they might not feel prepared to take the step because they cannot count on adequate resources to be successful. Consistent with this line of reasoning, Clark (2016) found in an in-depth study with successful and gritty individuals, that although grit was perceived as a necessary condition to succeed, it was not considered sufficient. This is consistent with the underlying mechanisms suggested by grit research of perseverance and long-term commitment to a goal. Gritty individuals might delay the decision to step into entrepreneurship until they perceive they have built the resources required to be successful. Our finding regarding the role of income level in the relationship between grit and entrepreneurship indicates that, indeed, when gritty individuals count on higher levels of resources they are more likely to create a business/become self-employed.

Wolfe and Patel (2016) found a positive interaction effect for grit and age of the entrepreneur, suggesting that grit constitutes a way of counteracting the resource limitations that young adults have in terms of human, social, and financial capital when involving in entrepreneurship. Wolfe and Patel’s results are not contradictory to our findings, as they compare the effect of grit in younger versus older adults, whereas our analyses focused strictly on young adults. Moreover, their sample was drawn from the general population from developing countries, where the resources for entrepreneurship are scarcer and the levels of necessary entrepreneurship are higher than in developed countries. In these adverse situations grit can be a more critical resource in the quest to make a living. Our sample, young adults from a developed country, excludes from the analysis entrepreneurship out of necessity as it considers that in such cases, the decision to engage in the entrepreneurial endeavor is determined by the lack of opportunities, not by a career choice, in which case the mechanisms at play should be different. Our study extends and refines therefore the results of Wolfe and Patel (2016) with a closer look at what happens within the group of young adults from a developed country, showing that gritty individuals will be more prone to set up a business/self-employ themselves when they can count on higher than current level of income and when they are dissatisfied with their current financial situation.

### Limitations

These results should be interpreted with caution, given the inherent limitations of our data set. First of all, our study uses cross-sectional data that do not allow us to trace the evolution in time of standing on the variables analyzed. Second, we used current level of income and satisfaction with one’s financial situation as proxies for the objective and subjective financial situation of the individual when he/she became involved in entrepreneurship. To dispel this situational specificity threat, we took several measures. We controlled in our models for individual’s economic situation at the age of 14 as an additional measure of individuals’ economic resources. Correlation analysis shows a positive and significant correlation between this variable and both objective and subjective current financial situation. This suggests that current measures of objective and subjective financial situation reflect to a certain extent the financial situation of the individual along his/her life course. Additionally, descriptive analyses of data from our sample show that 81.5% of the individuals who engaged in some form of entrepreneurship did so in the last 5 years before data collection. In such a short period of time it is unlikely that their level of income dramatically changed. Based on these data, we can conclude that the proxies we have used reflect with a certain degree of validity young adults’ general financial situation at the time they became involved in entrepreneurship.

Last, we would like to emphasize that other resource variables such as individuals’ social capital might be relevant for analyzing the translation of grit in entrepreneurial behavior. Future studies could explore thus the interaction between grit and social capital variables and its impact on individuals’ probability of becoming involved in entrepreneurship to get a deeper insight into the role that structural factors play in creating the context for grit to support behaviors that help with achievement of challenging long-term goals such as setting up a business.

## Conclusion

Overall, our results emphasize the importance of continuing to explore the association between grit and entrepreneurship and the factors that might moderate the association. Particularly, this study opens a new line of inquiry that seeks to gain a deeper comprehension of how young adult’s material and subjective circumstances influence the display of grit and its outcomes. The findings have implications for both grit and entrepreneurship research.

Regarding the former, grit has been studied mainly as a trait that can help people overcome adversity. Our results show that understanding grit’s role in the development of challenging and complex endeavors like entrepreneurship requires taking into account the resource endowments of the individual. People who are better equipped to capitalize on grit are the ones who have more resources (so face less adversity). This suggests that although grit can be an important and helpful life trait, there are other socio-economic factors that should be accounted for when study effects of grit and other individual differences.

Regarding the implications for entrepreneurship research, the study of contributions of individual differences to the prediction of entrepreneurship has ignored how the material conditions and individuals’ perceptions of these conditions may condition the predictive relationship. This study opens an interesting avenue for further research in this area.

## Ethics Statement

This study was carried out in accordance with the recommendations of the ethics committees of NetQuest and UPF with written informed consent from all subjects. All subjects gave written informed consent in accordance with the Declaration of Helsinki. The protocol was approved by the ethics committees of NetQuest and UPF.

## Author Contributions

JA-T and FF-M were responsible for the data gathering process as a part of a larger research project. JA-T and AB were responsible for doing the bibliographical search and setting up the hypotheses, doing the statistical analyses and reporting the results. RH supervised the data analysis process as well as the reporting process of the results particularly the proofreading of the paper in English.

## Conflict of Interest Statement

The authors declare that the research was conducted in the absence of any commercial or financial relationships that could be construed as a potential conflict of interest.
